# Factors Predicting the Frequency of Second Seizures in Patients Presenting to the Emergency Department With Seizures: A Prospective Observational Study

**DOI:** 10.7759/cureus.19271

**Published:** 2021-11-05

**Authors:** Öner Bozan, Şeref Emre Atiş, Bora Çekmen, Mücahit Şentürk, Mehmet Muzaffer İslam, Sevilay Ünver, Asim Kalkan

**Affiliations:** 1 Department of Emergency Medicine, Prof. Dr. Cemil Taşçıoğlu City Hospital, Istanbul, TUR; 2 Department of Emergency Medicine, Karabuk University Faculty of Medicine, Karabuk, TUR; 3 Department of Emergency Medicine, Prof. Dr. Cemil Taşcıoğlu City Hospital, Istanbul, TUR; 4 Department of Emergency Medicine, Umraniye Training and Research Hospital, Istanbul, TUR

**Keywords:** recurrent seizures, seizures, second seizures, epilepsy, emergency departments

## Abstract

Introduction

This study aimed to determine the factors that trigger seizures in patients reporting to our emergency department (ED) with seizures and the factors that affect recurrent seizures during the emergency department stay.

Materials and methods

This study was designed prospectively and was conducted among patients over the age of 18 years who reported to the ED of the Education and Research Hospital with complaints of epileptic seizure between July 01, 2020 and July 01, 2021. In addition to the sociodemographic information of the patients, the time of admission after the seizure, the medications used, comorbidities, the treatment given in the ED, history of trauma, previous epilepsy diagnosis, time of last seizure, alcohol use in the last 24 hours, insomnia, presence of infective symptoms in the past week, vital parameters, blood tests, and presence of recurrent seizure during hospital follow-up were recorded.

Results

The median age of the 102 patients included in the statistical analysis was 37 (25%-57%), and 61 (59.8%) were men. Patients who came to the ED with the complaint of seizures were divided into two groups, namely, those who had recurrent seizures and those who did not. When the differences between the groups in terms of various variables were examined, no statistically significant difference was found in the univariate analysis, except for WBC, aspartate aminotransferase (AST), and alanine aminotransferase (ALT) values. The diagnostic value of WBC, AST, and ALT levels in predicting recurrent seizures in emergency follow-up was analyzed using a receiver operating characteristic curve.

Conclusion

In this study, we could not find a parameter that can predict the probability of recurrent seizures in the ED in patients presenting with epileptic seizures.

## Introduction

Epilepsy is one of the most common neurological diseases that can be seen in all age groups. It requires a long period of follow-up and significantly reduces the quality of life [1,2]. An epileptic seizure is the occurrence of temporary signs and/or symptoms due to abnormal or synchronous neuronal activity in the brain. Symptoms vary depending on which areas of the brain are involved [3]. It has been reported that although seizure control can be achieved with medical treatment in 60-70% of the patients with epilepsy, the quality of life of the patients decreases and premature death rates increase [4]. Epilepsy, one of the most common neurological diseases, affects more than 70 million people worldwide [5].

About one-third of patients with epilepsy present to the ED every year [6]. This corresponds to one percent of the patients admitted to the ED [7]. Non-structural causes constitute approximately 50% of the seizure cases admitted to the ED, followed by vascular causes [6]. Among the causes that trigger epileptic seizures are electrolyte disorders, infections, insomnia, substance and alcohol use/withdrawal, medication change, skipping the drug dose, and irregular drug use or discontinuation of the drug [1].

This study aimed to determine the factors that trigger seizures in patients presenting to our ED with seizures and the factors that affect recurrent seizures during the stay in the ED.

## Materials and methods

This study was designed prospectively and was conducted among patients over the age of 18 years who applied to the ED of the Education and Research Hospital with complaints of epileptic seizure between July 01, 2020 and July 01, 2021. The study was initiated after approval from the Prof. Dr. Cemil Taşçıoğlu Ethics Committee (approval no: 48670771-514.10). Patients who were pregnant, did not have epileptic seizures, or had focal seizures were excluded from the study. The case form was filled by an emergency medicine assistant with >3 years of experience and an emergency medicine specialist. In addition to the sociodemographic information of the patients, the time of admission after the seizure, the medications used, comorbidities, the treatment applied in the ED, history of trauma, previous epilepsy diagnosis, time of last seizure, alcohol use in the last 24 hours, insomnia, presence of infective symptoms in the past week, vital parameters, blood tests, and presence of recurrent seizure during hospital follow-up were recorded. All seizures were witnessed by emergency medical services staff. The patient was followed up for four hours after his/her consciousness improved.

Statistical analysis

IBM SPSS Statistics for Windows, Version 26.0 (Released 2019. Armonk, NY: IBM Corp.) was used for statistical analysis. Conformity of the continuous data to normal distribution was analyzed with the Shapiro-Wilk test. Normally distributed data were expressed as mean (± standard deviation), whereas non-normally distributed data were expressed as median (25-75% quartile). Categorical data were expressed as frequency and percentage. Pairwise group comparisons were made with Mann Whitney U test for non-normally distributed continuous data. Student's t-test was used for pairwise group comparisons of normally distributed continuous data. Categorical data were compared between groups using the chi-square test, and Fisher’s exact test was used wherever necessary. The diagnostic value of statistically significant variables in predicting the probability of having a recurrent seizure in the ED was analyzed with the receiver operating characteristic (ROC) cure, and sensitivity, specificity, and accuracy values were calculated. P < 0.05 was accepted as statistically significant in all analyses.

## Results

The median age of the 102 patients included in the statistical analysis was 37 (25-57), and 61 (59.8%) were men. The main descriptive characteristics of the patients are summarized in Table 1.

**Table 1:** Descriptive characteristics of the patients GCS: Glasgow Coma Scale; COPD: Chronic obstructive pulmonary disease; CVE: Cerebrovascular event; CK: Creatine kinase; AST: Aspartate aminotransferase; ALT: Alanine aminotransferase; SpO2: Saturation of peripheral oxygen; CRP: c-reactive protein test; SD: Standard deviation

	Median (25%–75% quartiles) / N (%) / Mean (±SD)
Age	37 (25 - 57)
Sex (male)	61 (59.8%)
Systolic blood pressure (mmHg)	123 (±20)
Diastolic blood pressure (mmHg)	73 (±12)
Pulse (beats/min)	88 (±16)
SpO2 (%)	97 (96 - 98)
Body temperature (C)	36.7 (36.5 - 36.9)
Hypertension	10 (9.8%)
Diabetes mellitus	9 (8.8%)
COPD	3 (2.9%)
Prior ischemic CVE	13 (12.7%)
Malignancy	13 (12.7%)
Epilepsy	69 (67.6%)
WBC (10^3^/µL)	9.1 (6.4 - 11.2)
Hemoglobin (g/dl)	13.3 (12.1 - 14.8)
Platelet (10^3^/µL)	236 (195 - 301)
Glucose (mg/dL)	113 (95 - 144)
CRP (mg/L)	2.7 (1.1 – 10.5)
Urea (mg/dl)	27 (22 - 34)
Kreatinin (mg/dl)	0.72 (0.6 – 0.89)
Sodium (mmol/L)	138 (136 - 140)
Potassium (mmol/L)	4.16 (±0.55)
AST (U/L)	23 (18 – 33)
ALT (U/L)	17 (12 – 28)
CK (U/L)	109 (61 - 176)
Lactate (mmol/L)	2.77 (1.75 – 4.93)
Trauma history in the last week	5 (4.9%)
Alcohol intake in the last 24 hours	5 (4.9%)
Insomnia	5 (4.9%)
Emergency room admission within the first hour after the seizure	64 (62.7%)
History of seizures in the last month	57 (55.9%)
Diazepam administration in the emergency department	24 (23.5%)
Midazolam administration in the emergency department	17 (16.7%)
Levetiracetam administration in the emergency department	41 (40.2%)
Phenytoin administration in the emergency department	36 (35.3%)
Brain computed tomography	
Not performed	30 (29.4%)
No acute pathology	65 (63.7%)
Acute pathology	7 (6.9%)
Antiepileptic use	
No	26 (25.5%)
Regular Use	50 (49%)
Irregular use	26 (25.5%)
Seizure during emergency room follow-up	33 (32.4%)

Primary outcome measures

Patients who came to the ED with the complaint of seizures were divided into two groups, namely, those who had recurrent seizures and those who did not. When the differences between the groups were examined in terms of various variables, no statistically significant difference was found in the univariate analysis except for WBC, aspartate aminotransferase (AST), and alanine aminotransferase (ALT) values (p = 0.036, p = 0.006, p = 0.030, respectively). Univariate analysis results are summarized in Table 2.

**Table 2:** Univariate analysis results in terms of primary outcome * Patients who did not undergo brain CT were excluded from this analysis (patients with and without acute pathology in brain CT were compared). ԙ Those with no previous antiepileptic use were excluded from this analysis (patients using regular and irregular medication were compared). GCS: Glasgow Coma Scale, COPD: Chronic obstructive pulmonary disease, CVE: Cerebrovascular event, CK: Creatine kinase, AST: Aspartate aminotransferase, ALT: Alanine aminotransferase; CRP: c-reactive protein test; SD: Standard deviation

	Patients without recurrent seizures (mean 25%–75% quartiles/mean (± SD)/N (%))	Patients with recurrent seizures (mean 25%–75% quartiles/mean (± SD)/N(%))	p-value
Age	36 (25 - 58)	38 (26 - 50)	0.909
Sex (male)	39 (56.5%)	22 (66.7%)	0.327
Systolic blood pressure (mmHg)	123 (±19)	125 (±23)	0.513
Diastolic blood pressure (mmHg)	73 (±11)	76 (±15)	0.193
Pulse (beats/min)	87 (±16)	91 (±16)	0.321
sPO2 (%)	97 (96 - 98)	98 (96 - 98)	0.790
Body temperature (C)	36.7 (36.5 – 36.9)	36.8 (36.5 - 37)	0.341
Hypertension	7 (10.1%)	3 (9.1%)	0.587
Diabetes mellitus	6 (8.7%)	3 (9.1%)	0.606
COPD	2 (2.9%)	1 (3%)	0.695
Prior ischemic CVE	9 (13%)	4 (12.1%)	0.584
Malignancy	9 (13%)	4 (12.1%)	0.584
Epilepsy	50 (72.5%)	19 (57.6%)	0.133
WBC (10^3^/µL)	8.7 (6.1 – 10.1)	9.6 (7.7 – 13.2)	0.036
Hemoglobin (g/dl)	13.2 (11.9 – 14.8)	13.5 (12.3 – 14.8)	0.330
Platelet (10^3^/µL)	238 (199 - 299)	224 (190 - 309)	0.789
Glucose (mg/dL)	114 (96 - 136)	105 (94 - 168)	0.915
CRP (mg/L)	2.3 (1 – 8.3)	4.8 (1.2 – 16.8)	0.127
Urea (mg/dl)	27 (22 - 35)	28 (21 - 34)	0.786
Kreatinin(mg/dl)	0.72 (0.59 – 0.87)	0.68 (0.61 – 0.99)	0.718
Sodium (mmol/L)	138 (136 - 140)	139 (137 - 140)	0.118
Potassium (mmol/L)	4.16 (±0.47)	4.15 (±0.71)	0.940
AST (U/L)	22 (18 - 29)	28 (23 - 42)	0.006
ALT (U/L)	17 (12 - 26)	21 (14 - 41)	0.030
CK (U/L)	105 (62 - 171)	113 (55 - 215)	0.726
Lactate (mmol/L)	2.8 (1.8 – 4.4)	2.3 (1.7 – 6.7)	0.527
Trauma history in the last week	4 (5.8%)	1 (3%)	0.477
Alcohol intake in the last 24 hours	3 (4.3%)	2 (6.1%)	0.523
Insomnia	5 (7.2%)	0 (0%)	0.135
Emergency room admission within the first hour after the seizure	46 (66.7%)	18 (54.5%)	0.236
History of seizures in the last month	36 (52.2%)	21 (63.6%)	0.275
Presence of acute pathology in brain computed tomography *	3 (42.9%)	4 (57.1%)	0.184
Patients using regular antiepileptic drugs ^ԙ^	37 (67.3%)	13 (61.9%)	0.659

**Figure 1:**
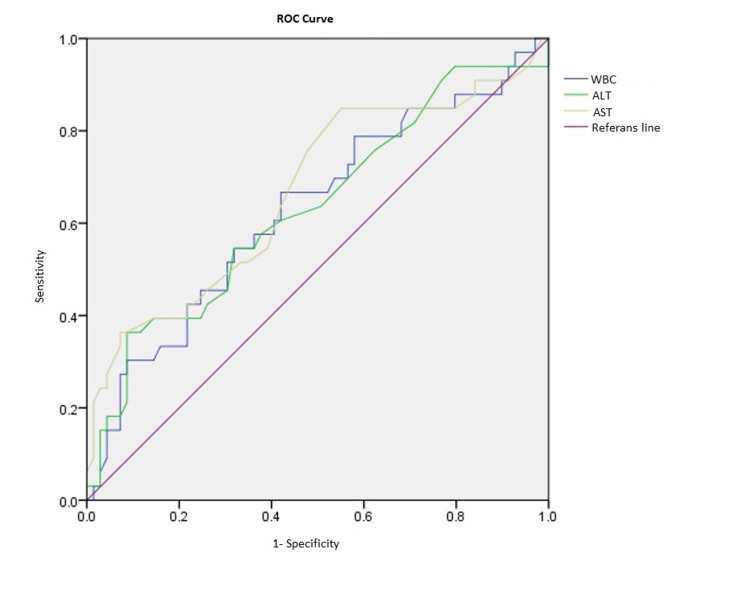
Receiver operating characteristic curve ROC: Receiver operating characteristic; AST: Aspartate aminotransferase; ALT: Alanine aminotransferase

The diagnostic value of WBC, AST, and ALT levels in predicting second seizures during emergency follow-up was analyzed using a ROC curve (Figure 1). Areas under the curve (AUC) were 0.628 (95% CI: 0.509-0.747), 0.668 (95% CI: 0.550-0.786), and 0.633 (95% CI: 0.514-0.753) for WBC, AST, and ALT, respectively. Diagnostic test performance criteria calculated for each of these variables according to the threshold values in which the sum of sensitivity and specificity is the highest are summarized in Table 3.

**Table 3:** Diagnostic test criteria for WBC, AST, and ALT calculated to predict recurrent seizures ALT: Alanine aminotransferase; AST: Aspartate aminotransferase, AUC: Area under the curve

	White sphere (threshold: 9.1)	AST (threshold: 21.5)	ALT (threshold: 33)
AUC (95% CI)	0.628 (95%CI: 0.509 - 0.747)	0.668 (95%CI: 0.550 – 0.786)	0.633 (95%CI: 0.514 – 0.753)
Sensitivity (95% CI)	66.7% (48,2% - 82%)	84,9% (68,1% - 95%)	36,4% (20,4% - 54,9%)
Specificity (95% CI)	58% (45,5% - 69,8%)	44,9% (32,9% - 57,4%)	91,3% (82,03% - 96,7%)
Positive likelihood ratio (95% CI)	1,6 (1,1 - 2,3)	1,5 (1,2 - 2)	4,2 (1,7 - 10,2)
Negative likelihood ratio (95% CI)	0,6 (0,3 - 1)	0,3 (0,2 - 0,8)	0,7 (0,5 - 0,9)
Positive predictive value (95% CI)	43,1% (34,4% - 52,2%)	42,4% (36,3% - 48,8%)	66,7% (45,2% - 82,9%)
Negative predictive value	78,4% (68,3% - 86%)	86,1% (72,6% - 93,5%)	75% (69,7% - 79,7%)
Accuracy	60,8% (50,6% - 70,3%)	57,8% (47,7% - 67,6%)	73,5% (63,9% - 81,8%)

## Discussion

This is the first study conducted to predict the recurrence of seizures in patients applying to the ED with epileptic seizures. In addition to the patients' blood parameters, vital signs at admission, time of last seizure, drug use, comorbidities, and sleep status were examined in the present study. WBC, AST, and ALT were found to be significant in the univariant analysis, but these differences were not clinically significant.

The incidence of epilepsy is slightly higher in men than in women [8,9]. However, there are studies showing the opposite, which may be due to the low number of patients [10]. In the present study, it was observed that the number of male patients was higher than the number of female patients, but this difference was not significant in predicting recurrent seizures in the ED. Similarly, the median age of the patients presenting with epilepsy was consistent with other studies in the literature [9].

A previous study showed that c-reactive protein (CRP) and WBC levels were significantly higher in patients presenting with seizures compared to the control group. However, this difference was not clinically significant [10]. In the present study in which seizure recurrence in the ED was examined, AST and ALT values were significant in addition to WBC. However, this difference is not clinically significant.

In studies performed with patients presenting with epileptic seizures, no significant relationship could be established between the types of drugs used for epilepsy and the frequency of seizures [9,11,12]. However, although there are studies showing that regular drug use reduces the frequency of seizures, there are also studies claiming the opposite [9,13]. In the present study, however, no relationship was found between regular drug use and having a second seizure in the ED.

Acute recurrent seizures are thought to present a risk of seizure-related neuronal damage comparable to that of prolonged seizures and require immediate medical attention [14]. It is not possible to predict this risk based on the parameters examined in the present study, including blood tests performed at the time of application, patient anamnesis, and clinical presentation. Therefore, we think that it is important to closely monitor the patients who present with epileptic seizures and to intervene whenever necessary.

This was a single-center study, and the number of patients was small. A multicenter study involving a large number of patients may provide more accurate results. In addition, different results can be obtained in studies conducted by distinguishing seizure types.

## Conclusions

In this study, we could not find a parameter that can predict the probability of recurrent seizures in the emergency department in patients presenting with seizures. Therefore, close follow-up of patients admitted to the ED with seizures is important in terms of morbidity and mortality.
